# Effect of milling procedures in CAD-CAM systems on the color changes of CAD-CAM polymethyl methacrylate resin material as interim material

**DOI:** 10.1186/s12903-024-04350-2

**Published:** 2024-05-28

**Authors:** Perihan Oyar, Mutahhar Ulusoy

**Affiliations:** 1 Department of Dental Prostheses Technology, Health Services Vocational High School, Hacettepe University, D Block, 3. Floor, Sihhiye, 06100 Ankara Turkey; 2 Department of Prosthodontics, Faculty of Dentistry, Near East University, Mersin, 10, Turkey

**Keywords:** Computer-aided design and computer-aided manufacturing, Polymethylmethacrylate, Color, Burs

## Abstract

**Purpose:**

This study aimed to investigate the effects of new and used burs on CAD-CAM PMMA resin color changes following thermocycling.

**Materials and methods:**

Twenty disk-shaped specimens (10 × 2 mm) were made using a single brand of CAD-CAM polymethyl methacrylate resin (Polident) for the color test. Group N consisted of half of the specimens that were machined using the new tungsten carbide bur set, and Group U consisted of the specimens that were milled using the used bur set (500 machining time). A color test was performed on the specimens both before and after thermocycling. For the statistical analysis, the Kruskal-Wallis and Dunn Pairwise Comparison tests were employed.

**Results:**

The ∆E* value of specimens (2.057) milled with the used bur was higher than those of specimens milled with the new bur (0.340), but this value is within clinically acceptable limits. After thermocycling, specimens milled with the utilized burs had the greatest L* (93.850) and b* (5.000) values. After thermocycling, statistically significant differences were discovered between Group N and Group U as well as between specimens milled with the utilized bur before and after thermocycling.

**Conclusion:**

Thermocycling process have an effect on the mean ∆E values of specimens milled with the used carbide bur, but these ∆E* values were not statistically significant.

**Clinical significance:**

The color and clinical performance of CAD-CAM restorations may be affected by variations in CAD-CAM milling bur properties, particularly those related to their frequent use.

## Background

Preliminary restorations have long been made with polymethyl methacrylate (PMMA). While this material has been able to produce adequate temporary functional restorations, it has come with disadvantages such shrinkage, excess monomer, and color changes [1, 2]. Dentists now have access to long-term temporary restorations made of polymer materials manufactured with CAD/CAM technology, as an alternative to traditionally polymerized materials. These so-called high-density polymers are polymerized in an industrial setting with optimum pressure and temperature parameters, under regulated and standardized conditions. Because of this, temporary restorations made using CAD/CAM technology from industrially produced resin blocks have a higher mechanical stability than restorations made using conventional methods [3, 4]. Since the prosthesis may be subjected to functional stress during implantation therapies like full occlusal reconstruction, periodontal surgeries, and maxillofacial rehabilitations, these restorations have the potential to be used for longer clinical periods [5–7]. The importance of aesthetic appeal of temporary polymer restorations increases with the length of time they are utilized in clinical settings. In order for dental restorations to be aesthetically pleasing, they need to closely mimic natural teeth [8]. In addition, one of the key components in fulfilling expectations of patients is the aesthetic appearance of the prosthesis [9–11]. and attaining outcomes in aesthetically demanding areas that are physiologically acceptable.

Denture materials must be enhanced to better resist temperature variations in the oral cavity in order to extend the life of dental restorations. Like all materials, long-term interim restorations are exposed to the oral environment and intraoral temperature changes [12]. 

After prolonged use, a variety of factors can influence the color change of denture materials. These factors has been widely studied [13–20], however the effects of tungsten carbide bur quality on the color changes of CAD-CAM PMMA resins have not been investigated. For this reason, the aim of the current study was to assess the impact of new and used burs on the color changes of CAD-CAM PMMA resin after and before thermocycling. The hypotheses were that color changes of CAD-CAM PMMA resin materials would affected by the use of new and used burs.

## Materials and methods

A single brand of CAD-CAM PMMA resin (PMMA CAD-CAM disc, multilayer; Polident d.o.o, Slovenija) was used to create a total of 20 disk-shaped specimens (10 × 2 mm) (*n* = 10/group). A CAD-CAM milling system (inlab MC X5, Sirona Dental System, GmbH, Germany) was used to mill the specimens. A total of ten disk-shaped specimens were machined using the new tungsten carbide bur sets (Dentsply, Slot 4, Bur 0.5 PMMA; Slot 5, Bur 1.0 PMMA; Slot 6, Bur 2.5 PMMA, Sirona), while the remaining ten were milled using the used burs, which had milled 500 PMMA crowns during 500 machining cycles.

CIE L*, a*, and b* [21] color values of all specimens were measured using a colorimeter EasyShade Advance (Vita Zahnfabrik, Bad Säckingen, Germany). L* a* b* values were measured at 3 different points on each specimen. Following that, these specimens were subjected to thermocycling (TC) (Thermocycler 1100/1200; SD Mechatronik, Germany) at 5000 cycles, 5 °C to 55 °C, 10 s transfer time, 30 s dwell time, and then color values of specimens were measured once more. Differences between the two color measurements (ΔE*) were calculated as a reference using the equation ΔE* (L, a,b)= [(L1 − L2)^2^+(a1 − a2)^2^+(b1 − b2)^2^]½ [21] where L1, a1 and b1 represent the values for the before thermal cycling and L2, a2 and b2 represent the values for the after thermal cycling. Clinically acceptable limits of the ΔE*value of specimens were evaluated as a reference using clinical color matching tolerance (Table 1) [22]. 

**Table 1:** Clinical color matching tolerance.

Color Difference (ΔE)	Clinical Color Match
0	Perfect
0.5 to 1	Excellent
1 to 2	Good
2 to 3.5	Clinically acceptable

The Kruskal-Wallis and Dunn Pairwise comparison tests were used to assess the color difference values, with *p*-values of less than.05 being deemed statistically significant.

## Results

Tables 2 and 3 displayed the mean values of L*, a*, and b*. Specimens milled with the used bur had a greater ΔE*value (2.057) than those milled with the new bur (0.340), although this difference is still within limits that are considered good/clinically acceptable (Table 4). After thermocycling, specimens milled with the utilized burs had the greatest L* (93.850) and b* (5.000) values. After thermocycling, statistically significant differences were discovered between Group N and Group U as well as between specimens milled with the utilized bur before and after thermocycling. There were no statistically significant variations in the a* values (Tables 2 and 3).

**Table 2:** Mean and standard deviation (SD) values of L*, a*, b* for specimens milled with the new bur

Specimens	L*		a*		b*	
	Mean	SD	Mean	SD	Mean	SD
**N/BTC**	92.930^a^	0.731	1.300^a^	0.094	4.470 ^a^	0.286
**N/ATC**	92.760^a^	0.804	1.380^a^	0.168	4.690 ^a^	0.296

Different superscript letters in each column indicate statistical difference (*p* < .05). N/BTC: Specimens milled with the new bur/before thermocycling; N/ATC: Specimens milled with the new bur/after thermocycling

**Table 3:** Mean and standard deviation (SD) values of L*, a*, b* for specimens milled with the used bur

Specimens	L*		a*		b*	
	Mean	SD	Mean	SD	Mean	SD
**U/BTC**	92.540^a^	1.384	1.270^a^	0.125	4.430 ^a^	0.275
**U/ATC**	93.850^b^	0.883	1.180^a^	0.204	5.000 ^b^	0.413

Different superscript letters in each column indicate statistical difference (*p* < .05). U/BTC: Specimens milled with the used bur/before thermocycling; U/ATC: Specimens milled with the used bur/after thermocycling

**Table 4:** Mean ΔE values and standard deviation (SD) of groups and clinical color matching tolerance

Groups	ΔE (Mean/ SD)	Clinical Color Match
**Specimens milled with the new bur**	0.340/0.695	Perfect
**Specimens milled with the used bur**	2.057/5.46	Clinically acceptable

## Discussion

Any color changes are among the most important clinical features of all dental materials. High color stability and resistance to color changes are desirable qualities for interim materials. High-pressure and temperature-polymerized PMMA resin blocks for CAD-CAM systems enhance optical characteristics and stability of color [23–25]. The current study investigated the effect of new and used burs on color changes of a CAD-CAM PMMA resin. Based on the findings, the hypothesis of the current study that new and used burs have an effect on color values of CAD-CAM PMMA specimens was partially rejected. In the current study L*, a* and b*values were increased in all specimens milled with the new tungsten carbide burs after thermocycling, but these were not statistically significant. However, statistically significant differences in L* and b* values were found in specimens milled with the used tungsten carbide burs. The color differences of specimens milled with the new tungsten carbide burs after thermocycling were clinically acceptable limits (ΔE*: 0.340). However, in specimens milled with the used tungsten carbide burs (ΔE*: 2.057), these values increased and, were within good/clinically acceptable (ΔE*<3.5) [22].

The Commission Internationale de l’Eclairage (CIE) L*a*b color system is a continuous color scale that encompasses all colors that are visible to the human eye. Therefore, it is suitable for research on how people perceive color variations in dental materials [23]. Worldwide, color research in dentistry is nearly solely conducted using the CIELAB color system. The CIE L*a*b* color scale is a roughly uniform color scale, meaning that variations in points displayed in the color space correlate to variations in colors as perceived visually. The CIE L*a*b* color space is arranged like a cube, with L* represented by the vertical axis. L* has values between 0 and 100, which indicate black and perfect reflecting diffuser, respectively [27]. The horizontal axes denoted as a* and b* correspond to the red-green and yellow-blue continuums, respectively. These continuums might have positive or negative values, and their numerical bounds are not specified [24]. 

The surface quality of interim restoration materials may be reduced and new surface defects may result from milling procedures [27]. Temperature changes and decreased resin surface quality can cause gradually color change of materials [30–32]. In the current study, particles of the milled CAD-CAM PMMA material may have gathered between the grooves of the bur with each use. Depending on how long and how often it was used, the surface of the bur may have worn. The surface of the bur may become worn from repeated use, which would reduce its ability to cut the surface of CAD-CAM PMMA material. This would also cause the milling quality of the bur to deteriorate and have an impact on the surface characteristics of material like surface roughness. The variation in the surface characteristics of CAD-CAM PMMA materials may have had an impact on the color qualities of materials. For this reason, in the current study, the specimens milled with the used bur may have exhibited the color changes after thermocycling.

For implant-supported prostheses, long-term interim restorations are needed following the immediate loading operation. These temporary restorations must tolerate prolonged occlusal loading and other oral environment factors, like as temperature variations, during the osseointegration process [31–34]. Long-term interim restorations should therefore have superior mechanical qualities and be able to endure the four to six month osseointegration and healing processes. As a result in the current study, the specimens were subjected to 5000 thermocycles, which corresponded to 6 months of physiological aging [35]. The samples milled using the used bur had a ΔE* value that was around six times higher than the ones milled with the new bur. The results of the current study suggested that, throughout the six months of use, the CAD-CAM PMMA temporary bridges milled with a used bur might potentially change color if they are subjected to acidic meals such as vinegar and colored beverages such as tea, coffee, and cola. This issue needs to be investigated with further studies.

Since this is one of the first research to look at how milling bur quality affects CAD-CAM PMMA resin color changes, the findings of current study cannot be compared to those of other studies. There has been a lot of study done on the properties of CAD-CAM resin materials, but not much has been done on the CAD-CAM systems themselves [12, 33, 36]. Whether the operators employed the burs in accordance with the manufacturer instructions or for a longer period of time was suspected to affect some properties of the CAD-CAM PMMA resin material. The results of this study should serve as a reminder that regular milling bur replacement is necessary to obtain satisfactory, consistent color outcomes for CAD-CAM PMMA resin. From an economic perspective, it is also critical to ascertain the duration during which CAD-CAM milling burs retain their cutting efficiency during the milling of different restorations.

The main purpose of this study was to investigate whether the bur quality affects the color change of the material. Therefore, we examined whether the use of used bur affects the color change of the PMMA. In our next study, we aim to see whether drinks with a coloring effect such as tea and coffee will cause color change in the PMMA material milled with the used bur. The current in vitro study had limitations in that it only evaluated one brand of CAD-CAM PMMA resin, only color changes of materials, and only one extent of milling using tungsten carbide burs (500 machining times). Future research should compare different resin materials, CAD-CAM systems, and milling procedures to better understand the effects of these variables and, ultimately, to improve the properties of CAD-CAM PMMA resin materials produced by CAD-CAM system.

## Conclusions

Within the limitations of the current study, the following results were drawn.

1. The L* values which indicate black and perfect reflecting diffuser and b*values which indicate yellow-blue continuums, of the tested interim materials with the used bur significantly increased after thermocycling.
2. The ∆E* value (2.057 ) which indicate color difference of specimens milled with the used bur was higher than those of specimens milled with the new bur (0.340), but this value is within clinically acceptable limits.

## Data Availability

The authors declare that the data supporting the findings of this study are available within the paper.
